# Applying the Clinician-reported Genetic testing Utility InDEx (C-GUIDE) to genome sequencing: further evidence of validity

**DOI:** 10.1038/s41431-022-01192-w

**Published:** 2022-10-04

**Authors:** Robin Z. Hayeems, Stephanie Luca, Anna C. E. Hurst, Meagan Cochran, Chelsea Owens, Alomgir Hossain, Lauren Chad, M. Stephen Meyn, Eleanor Pullenayegum, Wendy J. Ungar, David Bick

**Affiliations:** 1 Child Health Evaluative Sciences, The Hospital for Sick Children, Toronto, ON Canada; 2 Institute of Health Policy, Management and Evaluation, University of Toronto, Toronto, ON Canada; 3 University of Alabama at Birmingham, Birmingham, AL USA; 4 HudsonAlpha Institute for Biotechnology, Huntsville, AL USA; 5 Biostatistics, Design and Analysis Unit, The Hospital for Sick Children, Toronto, ON Canada; 6 Division of Clinical and Metabolic Genetics, The Hospital for Sick Children, Toronto, ON Canada; 7 Department of Pediatrics, University of Toronto, Toronto, ON Canada; 8 Department of Bioethics, The Hospital for Sick Children, Toronto, ON Canada; 9 Center for Human Genomics and Precision Medicine, The University of Wisconsin School of Medicine and Public Health, Madison, WI USA; 10 Dalla Lana School of Public Health, University of Toronto, Toronto, ON Canada; 11 Present Address: Genomics England, London, England

**Keywords:** Genetic testing, Outcomes research, Genetic testing

## Abstract

Genome sequencing (GS) outperforms other rare disease diagnostics, but standardized approaches to assessing its clinical utility are limited. This study assessed the validity of the Clinician-reported Genetic testing Utility InDEx (C-GUIDE), a novel tool for assessing the utility of genetic testing from a clinician’s perspective, for GS. C-GUIDE ratings were completed for patients who received GS results. For each patient, total C-GUIDE and single item global scores were calculated. Construct validity was assessed using linear regression to determine the association between C-GUIDE total and global item scores and measure the effects of potential explanatory variables. Ratings were completed for 67 pediatric and 36 adult patients. GS indications were neurological for 70.9% and results were diagnostic for 28.2%. When the C-GUIDE assessed primary (PV), secondary (SV), and pharmacogenomic (PGx) variants, on average, a one unit increase in the global item score was associated with an increase of 7.3 in the C-GUIDE score (*p* < 0.05). Diagnostic results were associated with an increase in C-GUIDE score of 5.0 compared to non-diagnostic results (*p* < 0.05) and an increase of one SV was associated with an increase of 2.5 (*p* < 0.05). For children, decreased age of one year was associated with an increase in C-GUIDE score of 0.3 (*p* < 0.05). Findings provide evidence that C-GUIDE measures the construct of clinical utility in pediatric and adult rare disease populations and is sensitive to changes in utility related to variant type. Quantifying the clinical utility of GS using C-GUIDE can inform efforts to optimize its use in patient care.

## Introduction

Genetic testing technology is rapidly evolving with the growth of precision medicine. While test evaluation typically relies on laboratory measures of performance (e.g. diagnostic yield) [1], tests can be costly, and analytically and ethically complex [2]. As such, a more comprehensive consideration of value and value for money is warranted to inform clinical adoption [3]. This is particularly true for genome sequencing (GS) where public and private health care systems are specifically seeking evidence of clinical utility to inform decisions about appropriate indications and timepoints for diagnostic testing [4, 5]. They are also adjudicating appropriate use of GS for the identification of secondary and/or pharmacogenomic findings [6–9]. To this end, a growing number of primary studies and evidence syntheses have emerged that report on various dimensions of clinical utility, inclusive of diagnostic, medical management, patient-reported, clinician-perceived, and health economic outcomes of GS [1, 10–13]. While these studies are continuing to better characterize the value and value for money of GS in a range of settings, standardized approaches to measuring the construct of clinical utility remain limited.

The Clinician-reported Genetic testing Utility InDEx (C-GUIDE^TM^) was developed to capture the informational value of genetic testing from a clinician’s perspective [14–16]. Specifically, C-GUIDE captures the utility of genetic testing as it relates to: (i) understanding diagnosis and prognosis, (ii) informing medical management, (iii) awareness and actionability of reproductive and health risks for patients and family members, and (iv) patient and family well-being. Modular in format, C-GUIDE items reflect these dimensions of value related to indication-based genetic test results (i.e. primary variants) and test results unrelated to the indication for testing (i.e. secondary and pharmacogenomic variants). A separate single item that reflects the overall value of the genetic test from the clinician’s perspective (i.e. global rating of utility) was also developed for the purpose of assessing C-GUIDE’s construct validity. In its first validation study at The Hospital for Sick Children (SickKids) in Toronto, Ontario, Canada, genetics professionals completed C-GUIDE ratings for 215 patients for whom genetic testing was performed for diagnostic purposes. The sample was largely pediatric and included utility ratings for patients with mostly neurodevelopmental phenotypes who received one of several different genetic test types (i.e. chromosome microarray, single gene, multi-gene panel). Findings indicated that an increase in the clinician’s global rating of utility was associated with an increase in C-GUIDE total score. In addition, partially/potentially diagnostic and non-diagnostic results had lower C-GUIDE scores than diagnostic results [14]. While these findings provide early evidence that C-GUIDE is a valid measure of clinical utility, insufficient patients were available to assess the validity of the C-GUIDE modules related to secondary and pharmacogenomic variants and insufficient patients were available to assess its validity in adults. Ongoing assessments of validity in different clinical settings for different types of genetic tests is therefore warranted.

The objective of this study was to assess the construct validity of all modules of C-GUIDE in two US-based tertiary-care genetics settings in which GS—and the reporting of secondary and pharmacogenomic variants—is more widely available.

## Materials and methods

### Settings

Genetics professionals based at The Smith Family Clinic for Genomic Medicine (SFC), HudsonAlpha Institute for Biotechnology in Huntsville, AL, USA and Children’s of Alabama (COA) in Birmingham, AL, USA participated. One genetic counsellor completed the C-GUIDE ratings at SFC and one medical geneticist completed the C-GUIDE ratings at COA after disclosing GS results to their patient or their patient’s family member. This study received ethics approval from the Institutional Review Boards that oversee SFC and COA (i.e. Western IRB Inc. and The University of Alabama at Birmingham, respectively) as well as the Research Ethics Board at SickKids. Clinician participants reviewed a consent document which indicated that survey completion constituted consent.

### Sample and recruitment

With instructions from the study team, clinician raters identified eligible patients, including outpatients for whom: (i) GS was completed as part of a diagnostic work up, and (ii) clinically validated positive or negative primary, secondary [17], and/or pharmacogenomic results [18] were reported directly to the patient or family up to two years prior to completing the C-GUIDE rating. Cases ineligible for C-GUIDE ratings included family members of probands who received cascade testing and cases for whom genetic testing was reported prenatally. To receive GS through SFC or COA, cases must have had a chronic medical condition with a likely genetic cause that remained unknown despite thorough evaluation. Candidates for GS were evaluated by a medical geneticist (ACEH at COA, DB at SFC) to ensure GS was an appropriate testing option.

All sequencing was performed at HudsonAlpha’s Clinical Services Lab (Huntsville, AL, USA). GS was performed with the Illumina NovaSeq 6000 sequencing platform and all primary, secondary, and complex indel variants were confirmed by Sanger sequencing. With respect to secondary variants, the laboratory reports findings in the following categories: untreatable childhood diseases (e.g., Tay-Sachs), treatable adult-onset diseases (e.g., Lynch syndrome), untreatable adult-onset diseases (e.g., autosomal dominant Alzheimer’s disease), and carrier status. As such, not all reported variants are considered to be medically actionable and patients could opt out of receiving them. Based on the reporting criteria used in this laboratory, ~3% of cases are identified to have variants in the American College of Medical Genetics (ACMG) secondary variants gene list (v2.0 [17, 19]) and approximately 33% are identified to have variants in genes related to other treatable conditions (e.g. *CHEK2, MITF, SPINK1, F2, TTR, NOD2)*. An additional 85% are identified to carry at least one autosomal recessive or X-linked disorder [9]. The pharmacogenomics report is restricted to variants that have been curated by PharmGKB as meeting the highest standard for evidence supporting a variant-drug association (category designated 1A or 1B). All PharmGKB variants in categories 1A or 1B that have dosing guidelines were included in the report except the CYP2D6*5 variant and variants in the *HLA-A*, *HLA-B*, and *CFTR* genes. A stratified recruitment approach was used to include approximately 35 cases for each result type (i.e. diagnostic/partially diagnostic, potentially diagnostic, non-diagnostic) across both sites.

### Data collection

Prior to data collection, the study team provided a 60 minute C-GUIDE training session for the clinician raters. C-GUIDE entries were completed between August 2020 and March 2021. For each rated patient, clinicians directly involved in the patient’s care completed a Case Description Questionnaire and C-GUIDE (Version 1.1, Supplementary Material) [14] through an online REDCap link [20, 21], using their knowledge of the patient, their own consult notes, or available medical records. Raters were able to start, stop, and resume data entry at their convenience. The Case Description Questionnaire included five items related to the index patient: age, sex, primary indication for testing, number of prior genetic tests, and test urgency and five items related to the test itself: GS strategy, result interpretation, disclosure modality, turnaround time, and time elapsed between result disclosure and C-GUIDE completion. GS strategies included singleton, duo, or trio; trios were defined as cases for whom the index case and two biological relatives received full GS. Result interpretations were defined as follows: *diagnostic* is a pathogenic/likely pathogenic variant that provides a complete explanation of phenotype; *partially diagnostic* is a pathogenic/likely pathogenic variant that provides a partial explanation of phenotype*; potentially diagnostic* is a variant of unknown significance that could provide a complete explanation of phenotype OR is a pathogenic/likely pathogenic variant in a recessive gene without a second hit; and *non-diagnostic* is a test result that provides no explanation of phenotype.

C-GUIDE includes modules to capture the utility of results related to the primary indication for testing (i.e. primary variants (PV) - 17 items), secondary variants (i.e. SV - 9 items), and pharmacogenomic variants (i.e. PGx - 4 items). Of the 30 C-GUIDE items, 27 are scored from 0 to 2 and three are scored from −2 to 0 using item-specific fixed response options. An item score >0 indicates positive utility, an item scores <0 indicates negative utility (“disutility”), and item scores of 0 indicate no utility. For each patient, three different total C-GUIDE scores were calculated (PV only, PV + SV, and PV + SV + PGx) by summing the item scores for each result type [15, 22]. Possible C-GUIDE total scores for each scoring strategy range from −2 to 32 for each PV, −4 to 48 for one PV plus one SV, and −6 to 54 for one PV plus one SV plus one PGx cluster. C-GUIDE is an ordinal scale and items are not weighted.

For the purpose of establishing construct validity of the C-GUIDE score, respondents completed a global rating of utility. This is a single ordinal item that asked raters to select whether, “when taking all results into account” (at the time of disclosure), the test “prompted better care”, “may prompt better care” or “did not change the care provided to the patient or his/her family.” For completion consistency, raters were instructed to define “better care” as a “change in care.” These fixed response options were assigned scores of 2, 1, and 0 respectively.

### Analysis

The assessment of construct validity (i.e. the degree to which a test measures what it claims to measure) [23] included three steps: (i) summarizing the case characteristics, (ii) testing hypotheses about the associations between C-GUIDE total scores and clinically important variables, and (iii) verifying relationships between C-GUIDE total scores and the global rating and between C-GUIDE total scores and clinically important variables using a regression model.

We summarized patient and test characteristics with descriptive statistics. Since clinical indication for testing was entered as free text, we developed a categorization scheme to organize these data. Category 1 included neurological indications and category 2 included non-neurological indications. Within category 1, patients were further classified according to the presence or absence of autism spectrum disorder (ASD), attention deficit hyperactivity disorder (ADHD), learning disability (LD), intellectual disability (ID), or developmental delay (DD). Patients in the non-ASD/ADHD/LD/ID/DD category were sub-categorized into five other neurological categories: adult neurological (e.g., neuropathy, spasticity, amyotrophic lateral sclerosis), hypotonia, seizures, hydrocephalus, or other. Within category 2, patients were sub-categorized as: immunological, postural orthostatic tachycardia syndrome (POTS)/dysautonomia, dysmorphic features, hematological, anomalous growth, or other.

Informed by the literature [1, 5, 9, 10, 24], local practice patterns, and study design considerations, we hypothesized that C-GUIDE total scores would rank as follows: (A) diagnostic/partially diagnostic results > potentially diagnostic results > non-diagnostic results; (B) trio GS > singleton/duo GS; (C) prior genetic testing > no prior genetic testing; (D) urgent tests > non-urgent tests; (E) younger patients > older patients; (F) presence of PV + SV + PGx > presence of PV + SV > presence of PV only. To test these hypotheses, a mean C-GUIDE score was calculated within each clinical sub-group defined by relevant patient characteristics. This was repeated for each of the three scoring strategies (PV only, PV + SV, PV + SV + PGx). The mean and range of C-GUIDE scores were determined for the patients associated with clinician global rating of “did,” “may,” or “did not” prompt a change in care. A Spearman coefficient of correlation was calculated between C-GUIDE total and global scores for each scoring strategy. Only complete questionnaires were included in the analysis. Detailed scoring and interpretation instructions can be found in the C-GUIDE User Manual [25].

As the data were right skewed (Kolmogorov-Smironov and Shapiro-Wilk tests were significant at *p* < 0.001), non-parametric tests were used to examine the relationship between clinically important variables and C-GUIDE scores. Mann–Whitney/Kruskal-Wallis tests were used to compare C-GUIDE scores calculated for each scoring strategy across diagnostic result categories, presence vs. absence of prior genetic testing, urgent vs non-urgent status, age categories, and clinical indications for testing. Descriptive statistics, scoring, and non-parametric analyses were completed in SPSS version 27 [26]. We used linear regression, with heteroscedasticity-consistent standard errors to account for the skewed data [27], to determine the association between C-GUIDE scores and global item scores along with other potentially explanatory variables. One model was fitted for each C-GUIDE scoring strategy (i.e. PV score, PV + SV score, PV + SV + PGx score). Sub-group analyses for children (≤18) and adults (>18) were performed. Adjusted R^2^ was used to select the most parsimonious model to fit the data. All modeling analyses were conducted in SAS software version 9.4 [28].

## Results

### Characteristics of patients and test results rated by C-GUIDE

A total of 103 patients were rated; one clinician at SFC completed 70 ratings and one clinician at COA completed 33 ratings (Table 1). For each patient, completing C-GUIDE took 5–10 min of clinicians’ time. A total of 67 pediatric and 36 adult patients were rated (mean age 19.9 years, SD = 21.8). The clinical indication for GS included a neurological phenotype for 70.9%, almost all tests were ordered non-urgently (98.1%), and the majority were singleton (82.5%). Of the rated patients, at least one diagnostic or partially diagnostic variant was identified in 28.2%, at least one potentially diagnostic variant was identified in 36.9%, and non-diagnostic results were reported in 35.0%. SV were identified in 95 patients (92.2%) and PGx were identified in 98 patients (95.1%). In 59.2% of patients, results were reported to the family within four months of test ordering. In 63.0% of patients, raters completed C-GUIDE within one week of reporting results to the family, and the majority of results were reported in person (68.0%; Table 1). Since only two patients were classified as urgent, this variable was removed from further analyses.

**Table 1:** Patient characteristics (*n* = 103).

	All Rated Patients *n* (%) (*n* = 103)
Age	
Mean (SD)	19.94 (21.78) years
Range	5 months – 84 years
0–2 years	22 (21.4)
3–10 years	29 (28.2)
11–18 years	16 (15.5)
19+ years	36 (35.0)
Sex	
Male	51 (49.5)
Female	52 (50.5)
Clinical indication for testing	
Neurological	73 (70.9)
ASD/ADHD/LD/ID/DD^†^	42 (40.8)
Non-ASD/ADHD/LD/ID/DD	31 (30.1)
Adult neurological^‡^	16 (15.5)
Hypotonia	5 (4.9)
Seizures	4 (3.9)
Hydrocephalus	3 (2.9)
Other, neurological	3 (2.9)
Non-neurological	30 (29.1)
Immunological	9 (8.7)
POTS^§^/Dysautonomia	6 (5.8)
Dysmorphic features	4 (3.9)
Dermatological	2 (1.9)
Hematological	2 (1.9)
Anomalous growth	2 (1.9)
Other, non-neurological	5 (4.9)
Number of prior genetic tests	
0	19 (18.4)
1	26 (25.2)
2	25 (24.3)
>2	33 (32)
Site**	
Site 1 (SFC)	70 (68.0)
Site 2 (COA)	33 (32.0)
Test Urgency^††^	
Urgent	2 (1.9)
Non-urgent	101 (98.1)
Genome Sequencing (GS) Strategy	
Singleton	85 (82.5)
Duo	5 (4.9)
Trio	13 (12.6)
Clinical interpretation of primary variant (PV1)	
Diagnostic/Partially diagnostic	29 (28.2)
Potentially diagnostic	38 (36.9)
Non-diagnostic/negative	36 (35.0)
Clinical interpretation of primary variant (PV2)	
Diagnostic/Partially diagnostic	4 (3.9)
Potentially diagnostic	13 (12.6)
Non-diagnostic/negative	11 (10.7)
Clinical interpretation of primary variant (PV3)	
Diagnostic/Partially diagnostic	0 (0)
Potentially diagnostic	5 (4.9)
Non-diagnostic/negative	5 (4.9)
Number of secondary variants identified (SV)	
0	8 (7.8)
1	13 (12.6)
2	18 (17.5)
3	19 (18.4)
4	19 (18.4)
5	15 (14.6)
6	7 (6.8)
7	4 (3.9)
Pharmacogenomics variants (PGx)	
Present	98 (95.1)
Absent/not requested	5 (4.9)
Result combinations (PV, SV, PGx)	
Potentially diagnostic PV + SV + PGx	33 (32.0)
Non-diagnostic/negative PV + SV + PGx	31 (30.1)
Diagnostic/partially diagnostic PV + SV + PGx	29 (28.2)
Non-diagnostic/negative PV + PGx	3 (2.9)
Diagnostic/partially diagnostic PV + PGx	2 (1.9)
Potentially diagnostic PV + SV	2 (1.9)
Non-diagnostic/negative PV only	2 (1.9)
Diagnostic/partially diagnostic PV only	1 (0.1)
Test turnaround time (TAT; date test ordered to date result disclosed to family)	
<4 months	66 (59.2)
≥4 months	37 (35.9)
C-GUIDE reporting interval (date result disclosed to family to date C-GUIDE completed)	
Same day	9 (8.7)
1–3 days	19 (18.4)
4 days–1 week	37 (35.9)
>1 week	38 (36.9)
Disclosure modality	
In person	70 (68.0)
By phone	22 (21.4)
Virtually (i.e. video-conference)	11 (10.7)

^†^*ASD* autism spectrum disorder, *ADHD* attention deficit hyperactivity disorder, *LD* learning disability, *ID* intellectual disability, *DD* developmental delay.

^‡^Examples of adult neurological: neuropathy, spasticity, amyotrophic lateral sclerosis (ALS).

^§^POTS: Postural Orthostatic Tachycardia Syndrome.

**SFC = The Smith Family Clinic for Genomic Medicine, COA = Children’s of Alabama.

^††^Urgency defined at clinician’s discretion (i.e. related to health state of index case and/or turnaround time).

### C-GUIDE scores and clinical characteristics

For PV, for 103 rated patients, the total C-GUIDE score ranged from −1 to 26 and the mean score was 6.2 (SD = 7.1). For PV + SV, the total score ranged from −1 to 35 and the mean score was 11.1 (SD = 7.9), and for the combination of PV + SV + PGx, the total score ranged from −1 to 36 and the mean score was 12.2 (SD = 8.0).

Table 2 presents mean C-GUIDE scores according to patient characteristics for each of the three scoring strategies: PV, PV + SV, and PV + SV + PGx. For all three scoring strategies, mean scores were higher among patients for whom diagnostic or partially diagnostic results were received compared to potentially diagnostic results (PV: 15.4 vs. 4.5, *p* < 0.001; PV + SV: 20.1 vs. 9.2, *p* < 0.001; PV + SV + PGx: 21.2 vs. 10.3, *p* < 0.001) or compared to non-diagnostic results (PV: 15.4 vs. 0.6, *p* < 0.001; PV + SV: 20.1 vs. 5.8, *p* < 0.001; PV + SV + PGx: 21.2 vs. 6.9, *p* < 0.001). For all three scoring strategies, mean scores were higher among patients who were 0–2 years old compared to 11–18 years old (PV: 9.3 vs. 2.8, *p* < 0.02; PV + SV: 13.3 vs. 6.4; *p* < 0.02, PV + SV + PGx: 14.6 vs. 7.5, *p* < 0.02). Scores were also higher among those who received GS results at COA where all patients were ≤18 years old and 82% had neurological indications for GS, compared to SFC where 51% of cases were 19+ years and 66% had neurological indications for GS (PV: 8.6 vs 5.1, *p* = 0.004; PV + SV: 13.2 vs. 10.1, *p* = 0.03; PV + SV + PGx: 14.8 vs. 11.0, *p* = 0.01).

**Table 2:** C-GUIDE scores and clinical characteristics.

	Mean PV^*^ C-GUIDE Score (SD) (*n* = 103)	Mean PV + SV^†^ C-GUIDE Score (SD) (*n* = 103)	Mean PV + SV + PGx^‡^ C-GUIDE Score (SD) (*n* = 103)
Patient age			
0–2 years (*n* = 22)	9.32 (8.40)^§*a*^	13.32 (8.32)**^d^	14.59 (8.29)^††*f*^
3–10 years (*n* = 29)	8.41 (7.32)^*b*^	12.21 (7.98)	13.52 (8.08)
11–18 years (*n* = 16)	2.81 (4.02)^*c*^	6.38 (5.77)^*e*^	7.50 (5.93)^*g*^
19+ years (*n* = 36)	4.14 (5.42)	10.89 (7.73)	11.78 (7.82)
Patient sex			
Male (*n* = 51)	7.49 (7.31)	12.29 (7.86)	13.55 (8.00)
Female (*n* = 52)	5.02 (6.64)	9.88 (7.79)	10.88 (7.78)
Clinical indication for testing			
Neurology (*n* = 73)	7.36 (7.50)^‡‡*h*^	11.53 (8.27)	12.67 (8.34)
Non-neurology (*n* = 30)	3.53 (4.99)^*i*^	9.97 (6.85)	11.07 (6.98)
Number of prior genetic tests			
0 (*n* = 19)	5.58 (6.59)	12.16 (8.08)	13.05 (8.17)
1 (*n* = 26)	4.92 (6.46)	9.92 (7.21)	11.00 (7.32)
2 (*n* = 25)	6.88 (7.40)	10.64 (7.08)	11.92 (7.15)
>2 (*n* = 33)	7.18 (7.60)	11.70 (8.99)	12.88 (9.08)
Site			
Site 1 (SFC; *n* = 70)	5.11 (6.66)^§§*j*^	10.06 (7.84)***^l^	10.96 (7.89)^†††*n*^
Site 2 (COA; *n* = 33)	8.64 (7.37)^*k*^	13.24 (7.64)^*m*^	14.85 (7.58)^*o*^
Test type			
Singleton (*n* = 85)	6.39 (7.02)	11.34 (7.39)	12.52 (7.46)
Duo/Trio (*n* = 18)	5.56 (7.38)	9.83 (10.04)	10.72 (10.16)
Result type for PV^‡‡‡^ (*n* = # variants)			
Diagnostic or Partially diagnostic (*n* = 33)	15.45 (5.67)^§§§*p*^	20.14 (6.55)****^s^	21.24 (6.59)^††††*v*^
Potentially diagnostic (*n* = 56)	4.53 (3.11)^*q*^	9.16 (5.06)^*t*^	10.34 (5.27)^*w*^
Non-diagnostic (*n* = 52)	0.64 (1.85)^*r*^	5.81 (4.36)^*u*^	6.89 (4.52)^*x*^
Number of secondary variants identified	-		
0 (*n* = 8)		6.88 (9.79)	7.50 (9.74)
1 (*n* = 13)		6.92 (5.56)^‡‡‡‡*y*^	8.08 (5.55)
2 (*n* = 18)		8.72 (6.64)	9.67 (6.76)
3 (*n* = 19)		11.53 (7.35)	12.74 (7.53)
4 (*n* = 19)		13.84 (8.09)	15.05 (8.00)
5 (*n* = 15)		11.00 (7.24)	12.47 (7.52)
6 (*n* = 7)		18.57 (9.05)^*z*^	19.57 (9.05)
7 (*n* = 4)		15.50 (5.20)	16.50 (5.20)
PGx variants (present vs absent)	-	-	
Present (*n* = 98)			12.43 (7.80)
Absent (*n* = 5)			7.80 (10.85)
Test turnaround time (TAT; date test ordered to date result disclosed to family)			
<4 months (*n* = 66)	6.29 (7.24)	10.79 (7.83)	11.92 (7.93)
≥4 months (*n* = 37)	6.16 (6.81)	11.59 (8.05)	12.70 (8.12)
C-GUIDE reporting interval (date result disclosed to family to date C-GUIDE completed)			
Same day (*n* = 9)	4.67 (5.10)	9.00 (5.50)	10.22 (5.83)
1–3 days (*n* = 19)	6.79 (8.22)	10.79 (8.90)	11.95 (9.08)
4 days – 1 week (*n* = 37)	6.38 (7.14)	11.92 (7.85)	12.97 (7.84)
>1 week (*n* = 38)	6.24 (7.06)	10.89 (8.02)	12.05 (8.13)
Disclosure modality			
In person or virtually (i.e., video-conference (*n* = 81)	7.15 (7.35)^§§§§*aa*^	11.95 (8.24)	13.14 (8.30)
By phone (*n* = 22)	2.91 (4.64)^*ab*^	7.86 (5.39)	8.77 (5.48)

*PV only = Includes all primary variants (1, 2, 3), as applicable. Mean is over all results.

^†^PV+SV = Includes all primary and secondary variants, as applicable. Mean is over all results.

^‡^PV+SV+PGx = Includes all primary, secondary and pharmacogenomic variant clusters, as applicable. Mean is over all results.

^§*a/b/c*^ Significant difference determined by Kruskal-Wallis, *p* = 0.001 between variable levels a & c, *p* < 0.05 and b & c, *p* < 0.05.

** *d/e* Significant difference determined by Kruskal-Wallis, *p* < 0.05 between variable levels d & e, *p* < 0.05.

^††*f/g*^ Significant difference determined by Kruskal-Wallis, *p* < 0.05 between variable levels f & g, *p* < 0.05.

^‡‡ *h/i*^ Significant difference determined by Mann–Whitney, *p* < 0.05 between variable levels h & i.

^§§ j/k^ Significant difference determined by Mann–Whitney, *p* < 0.01 between variable levels j & k.

*** *l/m* Significant difference determined by Mann–Whitney, *p* < 0.05 between variable levels l & m.

^†††*n/o*^ Significant difference determined by Mann–Whitney, *p* < 0.05 between variable levels n & o.

^‡‡‡^C-GUIDE ratings were completed on 141 primary variant findings reported in 103 patients.

^§§§*p/q/r*^ Significant difference determined by Kruskal-Wallis, *p* < 0.001 between variable levels p & q, p & r, and q & r, *p* < 0.001.

**** *s/t/u* Significant difference determined by Kruskal-Wallis, *p* < 0.001 between variable levels s & t, s & u, *p* < 0.001.

^†††† *v/w/x*^ Significant difference determined by Kruskal-Wallis, *p* < 0.001 between variable levels v & w, v & x, *p* < 0.001.

^‡‡‡‡ *y/z*^ Significant difference determined by Kruskal-Wallis, *p* = 0.05 between variable levels y & z, *p* < 0.05.

^§§§§ *aa/ab*^ Significant difference determined by Mann–Whitney, *p* = 0.005 between variable levels aa & ab.

For indication-related results only (i.e. PV), mean scores were higher among those with neurological phenotypes compared to those with non-neurological phenotypes (7.4 vs 3.5; *p* = 0.026), and higher among those who received GS results in-person or virtually compared to those who received results by phone (7.2 vs 2.9; *p* = 0.05). As expected, for scores related to PV + SV, mean scores were significantly higher among those who received a greater number of SV (PV + SV: 18.6 vs. 6.9; *p* = 0.04). Across all scoring strategies, mean scores did not differ significantly by sex, number of prior tests, GS strategy, turnaround time, or reporting interval (Table 2).

The global item score reflected the global clinical utility of all results rated per patient (i.e. PV + SV + PGx). Overall, where global item ratings indicated that test results prompted better care (*n* = 35), the mean C-GUIDE score was 19.6 (SD = 7.2; range: 7 to 36). Where global item ratings indicated that test results *may* prompt better care (*n* = 62), the mean C-GUIDE score was 8.5 (SD = 5.4; range: −1 to 26) and where global item ratings indicated that test results *did not* change care (*n* = 6), the mean C-GUIDE score was 7.2 (SD = 3.1; range: 2 to 10). The Spearman coefficients of correlation were 0.69 (*p* < 0.001) for PV only, 0.61 (*p* < 0.001) for PV + SV, and 0.63 for PV + SV + PGx (*p* < 0.001).

### C-GUIDE score as a function of clinical characteristics

As seen in Table 3, when the C-GUIDE score included only PV (Model 1), on average, a one unit increase in the global item score (i.e. moving from ‘did not prompt’ to ‘may prompt better care’ or from ‘may prompt better to care’ to ‘prompted better care’) was significantly associated with an increase of 6.5 in the C-GUIDE score (*p* < 0.05). In addition, diagnostic/partially diagnostic/potentially diagnostic results were associated with an increase of 5.5 in the C-GUIDE score compared to non-diagnostic results (*p* < 0.05).

**Table 3:** Associations between C-GUIDE score, global item score and clinical characteristics (*n* = 103).

	Model 1 PV		Model 2 PV + SV		Model 3 PV + SV + PGx	
Variable	Estimate	SE(HCC)^†^	Estimate	SE(HCC)	Estimate	SE(HCC)
Global item score	6.46*	1.01	7.10*	1.05	7.32*	1.05
Age	−0.01	0.02	0.06*	0.03	0.07*	0.03
Clinical indication	1.44	0.97	−0.01	1.23	−0.11	1.24
Genetic test number	−0.09	0.53	0.01	0.65	0.03	0.63
Primary variant result type: Diagnostic or Partially Diagnostic or Potentially Diagnostic vs Non-Diagnostic	5.50*	0.78	5.02*	0.96	4.96*	0.95
Number of Secondary Variants			2.38*	0.62	2.48*	0.6
Sex: Male vs Female	−0.09	1.08	0.69	1.27	0.71	1.25
GS Strategy: Singleton vs Duo/Trio	0.26	1.2	1.07	1.53	1.09	1.49
Site	0.93	1.4	1.47	1.51	2.11	1.46
Goodness-of-fit statistics: Adjusted R-square	59%		53%		56%	

^†^*HCC* heteroscedasticity-consistent standard errors.

*p < 0.05.

When the C-GUIDE score included PV + SV (Model 2), on average, a one unit increase in the global item score, representing a change from ‘did not prompt’ to ‘may prompt better care’ or ‘may prompt better to care’ to ‘prompted better care,’ was associated with an increase of 7.1 in the C-GUIDE score (*p* < 0.05). Across the whole sample, an increase in age of one year was associated with an increase in C-GUIDE score of 0.06 (*p* < 0.05). Diagnostic/partially diagnostic/potentially diagnostic results were associated with an increase of 5.0 in the C-GUIDE score compared to non-diagnostic results (*p* < 0.05), and every additional SV was associated with an increase in C-GUIDE score of 2.4 (*p* < 0.05).

When the C-GUIDE score included PV + SV + PGx (Model 3), results were similar to Model 2. On average, a one unit increase in the global item score was significantly associated with an increase of 7.3 in the C-GUIDE score (*p* < 0.05). An increase in age of one year was associated with an increase in C-GUIDE score of 0.07 (*p* < 0.05), diagnostic/partially diagnostic/potentially diagnostic results were associated with an increase of 5.0 in the C-GUIDE score compared to non-diagnostic results (*p* < 0.05), and an increase in the number of SV (by one) was associated with an increase in C-GUIDE score of 2.5 (*p* < 0.05).

Sub-group analyses for children and adults are presented in Table 4a and 4b. For children (≤18 years old), results aligned with the whole sample analyses except that a *decrease* in age of one year was associated with an increase in C-GUIDE score of 0.2 to 0.3 across the three models (*p* < 0.05; Table 4a). For adults (>18 years old), results aligned with the whole sample analyses, with the additional findings that an increase in age of one year was associated with an increase in C-GUIDE score of 0.1 for Model 3 (*p* < 0.05) and every additional prior genetic test was associated with an increase in C-GUIDE score of 1.4 for Model 1 (*p* < 0.05; Table 4b).

**Table 4:** a: Associations between C-GUIDE score, global item score and clinical characteristics for children (*n* = 67). b: Associations between C-GUIDE score, global item score and clinical characteristics for adults (*n* = 36).

a. Children	Model 1 PV		Model 2 PV + SV		Model 3 PV + SV + PGx	
Variable	Estimate	SE(HCC)^†^	Estimate	SE(HCC)	Estimate	SE(HCC)
Global item score	6.42*	1.05	7.12*	1.00	7.29*	0.99
Age	(−0.25)*	0.11	(−0.26)*	0.11	(−0.24)*	0.11
Clinical indication	0.52	1.69	−0.02	1.69	−0.11	1.66
Genetic test number	−0.45	0.67	−0.3	0.73	−0.31	0.69
Primary variant result type: Diagnostic or Partially Diagnostic or Potentially Diagnostic vs Non-diagnostic	5.26*	1.6	4.55*	1.13	4.59*	1.11
Number of Secondary Variants			1.67*	0.67	1.78*	0.64
Sex: Male vs Female	0.17	1.68	0.04	1.69	0.05	1.63
GS Strategy: Singleton vs Duo/Trio	0.13	1.63	0.61	1.71	0.53	1.66
Site	0.11	1.52	0.92	1.58	1.59	1.53
Goodness-of-fit statistics: Adjusted R-square	50%		60%			60%

b. Adults	Model 1 PV		Model 2 PV + SV		Model 3 PV + SV + PGx	
Variable	Estimate	SE(HCC)^†^	Estimate	SE(HCC)	Estimate	SE(HCC)
Global item score	6.19*	2.11	5.79*	2.83	6.14*	2.86
Age	0.02	0.02	0.10	0.05	0.11*	0.05
Clinical indication	1.33	0.96	−0.16	1.76	−0.31	1.79
Genetic test number	1.35*	0.6	1.78	1.07	1.76	1.08
Primary variant result type: Diagnostic or Partially Diagnostic or Potentially Diagnostic vs Non-Diagnostic	5.84*	0.72	6.01*	1.71	5.81*	1.72
Number of Secondary Variants			3.93*	0.84	3.99*	0.85
Sex: Male vs Female	0.21	1.05	1.93	1.94	1.93	1.95
GS Strategy: Singleton vs Duo/Trio	−0.32	1.42	0.67	1.99	0.81	1.97
Goodness-of-fit statistics: Adjusted R-square	70%		50%		50%	

^†^*HCC* heteroscedasticity-consistent standard errors.

*p < 0.05.

Clinical indication, sex, GS strategy, and site were not independently associated with unit changes in C-GUIDE scores in any of the fitted models.

## Discussion

Building on the preliminary validation study for C-GUIDE in Toronto, Canada [14], our findings provide further evidence that C-GUIDE measures the construct of clinical utility from the perspective of clinicians, in pediatric and adult rare disease populations. Specifically, the significant positive association between C-GUIDE total and global item scores in children and adults provides evidence of construct validity. Additional evidence of construct validity is illustrated by our finding that C-GUIDE scores aligned with a priori hypotheses that higher utility would be achieved among those for whom diagnostic results were received compared to those for whom non-diagnostic results were received, and among those for whom SV and PGx were identified compared to those for whom they were not. Moreover, the magnitude of the effects of result type (i.e. diagnostic) and the presence of SV and/or PGx are similar.

In the overall analysis (children and adults combined), when SV and PGx were included in the C-GUIDE score (Table 3 - Models 2, 3), increased age was associated with higher C-GUIDE scores. While some SV indeed have medical management implications (i.e. clinical utility) for children [8], many SV are more likely to be medically actionable in adulthood [29]. Additionally, where SV reveal carrier status (i.e. in contrast with medically actionable variants), the potential utility of these findings is likely to be greater among individuals who have reached adulthood and may be interested in family planning. While the specific type of SV was not specified for the cases rated by C-GUIDE in this study, ~85% of SV identified by HudsonAlpha’s Laboratory were associated with carrier status [9]. As such, it is reasonable to speculate that the utility of carrier status for family planning provides some explanation for the observed age effect. With respect to PGx, we speculate that the observed increase in C-GUIDE score with age reflects greater use of prescription medication with age [30], thereby increasing the utility of PGx. As expected, a greater number of reported SV and PGx was associated with higher utility scores.

Our sub-group analysis by age refines our interpretation of the observed effect of age. For children alone, we see the effect of age in the opposite direction. That increased age was associated with decreased C-GUIDE scores for all scoring strategies aligns with arguments in the literature that favour the use of GS as early as possible in the diagnostic journey for patients with rare disease [24, 31, 32]. We speculate that for children, the utility of a timely diagnosis trumps the utility of SV and PGx. Interestingly, for adults, we observed that the receipt of prior genetic testing was positively assicated with C-GUIDE scores, perhaps suggesting that the utility of early diagnosis for adults may not be as pronounced as it is for children. Finally, the absence of an observed effect for clinical indication, sex, and GS strategy suggests that the utility of GS results is independent of these factors.

The mean and range of C-GUIDE scores associated with each global item response option were similar to those reported in the Toronto validation study [14]. As expected, C-GUIDE scores in the present study were higher among patients with the highest global rating and lower among patients with the lowest global rating. While C-GUIDE and global scores are moderately correlated (r = 0.61−0.69), C-GUIDE ranges for each global item category overlapped, suggesting that more work is required to establish the clinical meaning of various C-GUIDE scores as well as population norms and thresholds for high and low utility ratings for different categories of variants. As discussed in the first C-GUIDE validation study, as the list of reportable PV, SV and PGx expands and as variant interpretations change with an evolving knowledge base, C-GUIDE scores, and their clinical meaning, will evolve [14].

We acknowledge limitations related to this assessment of construct validity. First, small numbers of patients were enrolled for some categories of clinical characteristics. As such, hypotheses regarding the effects of these characteristics on C-GUIDE score could not be tested. For example, the low rate of urgent patients (i.e. 2%) precluded our ability to assess the hypothesis that greater clinical utility would be achieved for urgent testing compared to non-urgent testing. Second, small sample size precluded the assessment of plausible interactions. Third, while sub-group analyses by age demonstrated construct validity in both children and adults and refined our interpretation of the observed age effect, further validity testing in adults is warranted given the small number of adults included. Fourth, while we were able to demonstrate construct validity related to SV and PGx, we could not differentiate the utility of medically actionable versus non-medically actionable SV since raters were not asked to characterize the type(s) of SV identified. Finally, ratings provided for the retrospective cases at SFC reflect the content of the clinician’s consult note that was written up to two years prior to C-GUIDE completion, rather than the perspective of the clinician at the time of result disclosure. To what extent real-time versus retrospective assessments of utility differ warrants further study.

Validating C-GUIDE in a clinical setting that is external to the environment in which it was developed further demonstrates its credibility and generaliability as an empiric universal strategy for measuring the value of genetic testing, as perceived by clinicians. Moreover, the application of C-GUIDE to the context of GS enabled us to assess its validity for SV and PGx, an emerging area of practice that warrants robust approaches to evidence development. Further work to validate its use among non-genetics providers, to understand if/how utility ratings change over time, and how patients orient to the concept of utility is ongoing. In conclusion, our findings suggest that C-GUIDE achieves acceptable levels of construct validity and can be integrated into studies that aim to capture the clinical utility of a broad range of genetic tests used for the purpose of establishing a genetic diagnosis in children and adults. Quantifying clinical utility in standardized ways will inform efforts to optimize the use of genetic and genomic technologies in patient care and provides key evidence for funding and adoption decisions.

## Supplementary information

Supplementary Material

## Data Availability

All data generated or analyzed during this study are either included in this published article or available upon request.
