# Supplementary material for: Applying the Clinician-reported Genetic testing Utility InDEx (C-GUIDE) to genome sequencing: further evidence of validity
Source: Eur J Hum Genet. 2022 Oct 4;30(12):1423–31. doi: 10.1038/s41431-022-01192-w (PMC9712646; doi:10.1038/s41431-022-01192-w)
Supplement: Supplementary file 1 — Supplementary Material [file 41431_2022_1192_MOESM1_ESM.docx]

# Supplementary Material

# C-GUIDE^TM^ Version 1.1 (not recommended for future use)

**C-GUIDE Section 1: Results related to primary indication for testing**

| **Item** | **Response Options** |
| --- | --- |
| ***The genetic testing that my patient had…*** | |
| 1. Provided a genetic explanation for my patient's health condition | - Provided a COMPLETE genetic explanation - Provided a PARTIAL genetic explanation - Provided a POSSIBLE genetic explanation - Provided NO genetic explanation |
| 1. Reduced the likelihood of other differential diagnoses | - COMPLETELY REDUCED the likelihood of other differential diagnoses - PARTIALLY REDUCED the likelihood of other differential diagnoses - DID NOT REDUCE the likelihood of other differential diagnoses |
| 1. Provided information about the natural history of or medical issues associated with my patient's condition | - Provided SIGNIFICANT information about the natural history of or medical issues associated with my patient's condition - Provided SOME information about the natural history of or medical issues associated with my patient's condition - Provided NO information about the natural history of or medical issues associated with my patient's condition |
| 1. Indicated that further testing to identify a genetic diagnosis can be avoided | - Indicated that further testing to identify a genetic diagnosis CAN BE AVOIDED - Indicated that further testing to identify a genetic diagnosis MAY STILL BE REQUIRED, now or in the future |
| 1. Indicated that previous surveillance or monitoring related to my patient’s condition can be discontinued or avoided | - Indicated that previous surveillance/monitoring can be DISCONTINUED OR AVOIDED - Indicated that previous surveillance/monitoring is STILL REQUIRED - Previous surveillance/monitoring is NOT RELEVANT to this case |
| 1. Facilitated my patient's access to or continuation of a community or educational service (e.g. learning, rehabilitation resources) that would not have been available without the testing | - FACILITATED access to or continuation of a community or educational service - DID NOT FACILITATE access to or continuation of a community or educational service |
| 1. Enabled me to identify and access a clinical trial that I wouldn't have been able to access without the testing | - ENABLED me to IDENTIFY and ACCESS a clinical trial - ENABLED me to IDENTIFY a clinical trial - DID NOT ENABLE me to identify or access a clinical trial |
| 1. Enabled me to identify a support group for my patient or his/her family that I wouldn’t have considered without the testing | - ENABLED me to identify a support group - DID NOT ENABLE me to identify a support group |
| 1. Prompted a referral or investigation for the purpose of surveillance or monitoring that would not have been prompted on clinical grounds | - PROMPTED a referral or investigation for surveillance/monitoring - PROMPTED a referral or investigation for surveillance/monitoring that MAY NOT BE NECESSARY (e.g. variant of uncertain significance) - DID NOT PROMPT a referral/investigation for surveillance/monitoring |
| 1. Provided information to guide medication management | - GUIDED current medication management - MAY GUIDE medication management in the future - DID NOT PROVIDE information that would guide medication management, now or in the future |
| 1. Provided information about surgical management | - ENABLED a discussion or offer of a surgical option - AVOIDED a discussion or offer of a surgical option - A surgical option is NOT RELEVANT at this time or NOT RELATED to the genetic test results |
| 1. Provided information about a contraindicated behaviour (e.g. competitive sports) | - ENABLED me to provide information about a contraindicated behaviour - Information about a contraindicated behaviour is NOT RELEVANT at this time |
| 1. Provided recurrence risk information for my patient | - Provided recurrence risk information that is RELEVANT to my patient at this time - Provided recurrence risk information that MAY BE RELEVANT to my patient in the future - Cannot be determined (e.g. variant of uncertain significance, did not provide information) |
| 1. Provided recurrence risk information for my patient’s family | - Provided recurrence risk information that is RELEVANT to my patient’s family at this time - Provided recurrence risk information that MAY BE RELEVANT to my patient’s family in the future - Cannot be determined (e.g. variant of uncertain significance, family member(s) did not receive testing or unknown if tested) |
| 1. Clarified potential health risks for my patient’s family | - CLARIFIED potential health risks for my patient’s family - DID NOT CLARIFY health risks for my patient’s family - Cannot be determined (e.g. variant of uncertain significance, family member(s) did not receive testing or unknown if tested) |
| 1. Generated psychosocial benefit for my patient or his/her family | - SIGNIFICANT psychosocial benefit was experienced - MODERATE psychosocial benefit was experienced - NO psychosocial benefit was experienced - Cannot be determined |
| 1. Generated psychosocial concern for my patient or his/her family | - SIGNIFICANT psychosocial concern was experienced - MODERATE psychosocial concern was experienced - NO psychosocial concern was experienced - Cannot be determined |

**C-GUIDE Section 2: Secondary Variants**

Did you disclose SECONDARY variant results?

- Yes
- No

N.B. For the purpose of this index, secondary variants include medically actionable variants unrelated to the indication for testing.

| **Item** | **Response Options** |
| --- | --- |
| ***The genetic testing that my patient had…*** | |
| 1. Prompted a referral or investigation for the purpose of surveillance or monitoring that would not have been prompted on clinical grounds | - PROMPTED a referral or investigation for surveillance/monitoring - PROMPTED a referral or investigation for surveillance/monitoring that MAY NOT BE NECESSARY (e.g. variant of uncertain significance) - DID NOT PROMPT a referral/investigation for   surveillance/monitoring |
| 2. Provided information to guide medication management | - GUIDED current medication management - MAY GUIDE medication management in the future - DID NOT PROVIDE information that would guide medication management, now or in the future |
| 3. Provided information about surgical management | - ENABLED a discussion or offer of a surgical option - AVOIDED a discussion or offer of a surgical option - A surgical option is NOT RELEVANT at this time or NOT RELATED to the genetic test results |
| 4. Provided information about a contraindicated behaviour (e.g. competitive sports) | - ENABLED me to provide information about a contraindicated behaviour - Information about a contraindicated behaviour is NOT RELEVANT at this time |
| 5. Provided recurrence risk information for my patient | - Provided recurrence risk information that is RELEVANT to my patient at this time - Provided recurrence risk information that MAY BE RELEVANT to my patient in the future - Cannot be determined (e.g. variant of uncertain significance, did not provide information) |
| 6. Provided recurrence risk information for my patient's family | - Provided recurrence risk information that is RELEVANT to my patient's family at this time - Provided recurrence risk information that MAY BE RELEVANT to my patient's family in the future - Cannot be determined (e.g. variant of uncertain significance, family member(s) did not receive testing or unknown if tested) |
| 7. Clarified potential health risks for my patient's family | - CLARIFIED potential health risks for my patient's family - DID NOT CLARIFY health risks for my patient's family - Cannot be determined (e.g. variant of uncertain significance, family member(s) did not receive testing or unknown if tested) |
| 8. Generated psychosocial benefit for my patient or his/her family | - SIGNIFICANT psychosocial benefit was experienced - MODERATE psychosocial benefit was experienced - NO psychosocial benefit was experienced - Cannot be determined |
| 9. Generated psychosocial concern for my patient or his/her family | - SIGNIFICANT psychosocial concern was experienced - MODERATE psychosocial concern was experienced - NO psychosocial concern was experienced - Cannot be determined |

**C-GUIDE Section 3: Pharmacogenomic Variants**

Did you disclose PHARMACOGENOMIC results?

- Yes
- No

N.B. For the purpose of this index, pharmacogenomic results include those that are identified through a targeted pharmacogenomic analysis and could be relevant to medication management now or in the future.

If yes, please complete C-GUIDE once for the pharmacogenomic result(s) disclosed. For the purpose of this study, pharmacogenomic results are typically disclosed as a ‘cluster’ of variants to the patient or family.

| **Item** | **Response options** |
| --- | --- |
| ***The genetic testing that my patient had…*** | |
| 1. Provided information to guide medication management for my patient | - GUIDED current medication management - MAY GUIDE medication management in the future - DID NOT PROVIDE information that would guide medication management, now or in the future |
| 1. Provided information to guide medication management for my patient’s family | - GUIDED current medication management for my patient’s family - MAY GUIDE medication management for my patient’s family in the future - DID NOT PROVIDE medication management information for my patient’s family, now or in the future - Cannot be determined (e.g. variant of uncertain significance, family member(s) did not receive testing or unknown if tested) |
| 1. Generated psychosocial benefit for my patient or his/her family | - SIGNIFICANT psychosocial benefit was experienced - MODERATE psychosocial benefit was experienced - NO psychosocial benefit was experienced - Cannot be determined |
| 1. Generated psychosocial concern for my patient or his/her family | - SIGNIFICANT psychosocial concern was experienced - MODERATE psychosocial concern was experienced - NO psychosocial concern was experienced - Cannot be determined |

**C-GUIDE Section 4: Global item**

| Taking into account all of the results you have just rated for this test, the genetic testing that my patient had | - Prompted better care for my patient or his/her family - May prompt better care for my patient or his/her family in the future - Did not change the care provided to my patient or his/her family |
| --- | --- |

© Copyright 2021, THE HOSPITAL FOR SICK CHILDREN.

#

# C-GUIDE^TM^ Version 1.2 (recommended for future use)

The Clinician-reported Genetic testing Utility InDEx (C-GUIDE)^TM^ aims to capture the clinical utility of genetic testing once results are disclosed to patients/families, from the perspective of the ordering clinician.

C-GUIDE includes (i) 17 C-GUIDE items related to results received for the primary indication for testing, and if applicable, (ii) 4-9 C-GUIDE items related to secondary or pharmacogenomic variant results received.

Thinking about the result(s) you just disclosed related to the primary indication for testing, please complete the following:

N.B. If you disclosed multiple results from the same test, please complete the C-GUIDE once for each result disclosed. You will be prompted to do this after you complete C-GUIDE for the first result. If you disclosed secondary or pharmacogenomic results from this test, you will be asked about those specific results later.

**C-GUIDE: Core Index**

| **Item** | **Response Options** |
| --- | --- |
| ***The genetic testing that my patient had…*** | |
| 1. Provided a genetic explanation for my patient's health condition | - Provided a COMPLETE genetic explanation **[2]** - Provided a PARTIAL genetic explanation **[1]** - Provided a POSSIBLE genetic explanation **[1]** - Provided NO genetic explanation **[0]** |
| 1. Reduced the likelihood of other potential diagnoses in my differential | - COMPLETELY REDUCED the likelihood of other potential diagnoses in my differential **[2]** - PARTIALLY REDUCED the likelihood of other potential diagnoses in my differential **[1]** - DID NOT REDUCE the likelihood of other potential diagnoses in my differential **[0]** - Not applicable **[0]** |
| 1. Provided information about the natural history of or medical issues associated with my patient's condition | - Provided SIGNIFICANT information about the natural history of or medical issues associated with my patient's condition **[2]** - Provided SOME information about the natural history of or medical issues associated with my patient's condition **[1]** - Provided NO information about the natural history of or medical issues associated with my patient's condition **[0]** |
| 1. Indicated that further testing to identify a genetic diagnosis can be avoided | - Indicated that further testing to identify a genetic diagnosis CAN BE AVOIDED **[2]** - Indicated that further testing to identify a genetic diagnosis MAY STILL BE REQUIRED, now or in the future **[0]** |
| 1. Indicated that previous surveillance or monitoring related to my patient’s condition can be discontinued or avoided | - Indicated that previous surveillance/monitoring can be DISCONTINUED OR AVOIDED **[2]** - Indicated that previous surveillance/monitoring is STILL REQUIRED **[0]** - Previous surveillance/monitoring is NOT RELEVANT to this case **[0]** |
| 1. Facilitated my patient's access to or continuation of a community or educational service (e.g. learning, rehabilitation resources) that would not have been available without the testing | - FACILITATED access to or continuation of a community or educational service **[2]** - DID NOT FACILITATE access to or continuation of a community or educational service **[0]** |
| 1. Enabled me to identify and access a research study that I wouldn’t have been able to access without the testing | - ENABLED me to IDENTIFY and ACCESS a clinical trial **[2]** - ENABLED me to IDENTIFY a clinical trial **[1]** - Enabled me to IDENTIFY and/or ACCESS a natural history or functional study to assist with result interpretation **[1]** - DID NOT ENABLE me to IDENTIFY or ACCESS a clinical trial, natural history or functional study **[0]** |
| 1. Enabled me to identify a support group for my patient or his/her family that I wouldn’t have considered without the testing | - ENABLED me to identify a support group **[2]** - DID NOT ENABLE me to identify a support group **[0]** |
| 1. Prompted a referral or investigation for the purpose of surveillance or monitoring that would not have been prompted on clinical grounds | - PROMPTED a referral or investigation for surveillance/monitoring **[2]** - PROMPTED a referral or investigation for surveillance/monitoring that MAY NOT BE NECESSARY (e.g. variant of uncertain significance) **[1]** - DID NOT PROMPT a referral/investigation for surveillance/monitoring **[0]** |
| 1. Provided information to guide medication management | - GUIDED current medication management **[2]** - MAY GUIDE medication management in the future **[1]** - DID NOT PROVIDE information that would guide medication management, now or in the future **[0]** |
| 1. Provided information about surgical management | - ENABLED a discussion or offer of a surgical option **[2]** - AVOIDED a discussion or offer of a surgical option **[1]** - A surgical option is NOT RELEVANT at this time or NOT RELATED to the genetic test results **[0]** |
| 1. Provided information about a contraindicated behaviour (e.g. competitive sports) | - ENABLED me to provide information about a contraindicated behaviour **[2]** - Information about a contraindicated behaviour is NOT RELEVANT at this time **[0]** |
| 1. Provided recurrence risk information for my patient | - Provided recurrence risk information that is RELEVANT to my patient at this time **[2]** - Provided recurrence risk information that MAY BE RELEVANT to my patient in the future **[1]** - Cannot be determined (e.g. variant of uncertain significance, did not provide information) **[0]** |
| 1. Provided recurrence risk information for my patient’s family | - Provided recurrence risk information that is RELEVANT to my patient’s family at this time **[2]** - Provided recurrence risk information that MAY BE RELEVANT to my patient’s family in the future **[1]** - Cannot be determined (e.g. variant of uncertain significance, family member(s) did not receive testing or unknown if tested) **[0]** |
| 1. Clarified potential health risks for my patient’s family | - CLARIFIED potential health risks for my patient’s family **[2]** - DID NOT CLARIFY health risks for my patient’s family **[0]** - Cannot be determined (e.g. variant of uncertain significance, family member(s) did not receive testing or unknown if tested) **[0]** |
| 1. Generated psychosocial benefit for my patient or his/her family | - SIGNIFICANT psychosocial benefit was experienced **[2]** - MODERATE psychosocial benefit was experienced **[1]** - NO psychosocial benefit was experienced **[0]** - Cannot be determined **[0]** |
| 1. Generated psychosocial concern for my patient or his/her family | - SIGNIFICANT psychosocial concern was experienced **[-2]** - MODERATE psychosocial concern was experienced **[-1]** - NO psychosocial concern was experienced **[0]** - Cannot be determined **[0]** |

**Guidance for Raters:**

**Item 3:** This includes gaining insight about natural history by way of reverse phenotyping that may be prompted by genetic test results. Reverse phenotyping refers to the identification of clinical features based on genotype.

**Item 6:** This refers to whether genetic testing results theoretically facilitated access to services, not if results actually facilitated access to services. Due to school district specific policies, the final outcome may be unclear.

**Item 11:** This refers to whether genetic testing results provided information about surgical management, specifically. It does not refer to a situation where surgery was considered for diagnostic reasons (e.g. muscle biopsy).

**Item 13:** The recurrence risk information is considered relevant if the patient is of reproductive age and is considering having children now or in the near future (i.e. within 1 year).

**Item 14:** Family includes parents, siblings and extended family. The recurrence risk information is considered relevant if the patient’s family member is of reproductive age and is considering having children now or in the near future (i.e. within 1 year).

**Item 15:** Reduction of risk counts. For example, if there was a question that other family members could have the same condition, but the primary finding was de novo, there would be a reduction of risk for a family member.

**Items 16/17:** If you do not have a clear memory of the session or did not record psychological response in clinic notes, choose the ‘cannot be determined’ response option.

**Items 5-7, 9-13:** Not applicable when the proband is deceased. In this case, item should be left blank.

**C-GUIDE: Secondary Variants Index**

Did you disclose SECONDARY variant results?

- Yes
- No

N.B. For the purpose of this index, secondary variants include medically actionable variants unrelated to the indication for testing.

If yes, please complete a C-GUIDE once for each secondary result disclosed to the patient or family.

| **Item** | | **Response options** |
| --- | --- | --- |
| ***The genetic testing that my patient had…*** | | |
| 1. Prompted a referral or investigation for the purpose of surveillance or monitoring that would not have been prompted on clinical grounds | - PROMPTED a referral or investigation for surveillance/monitoring **[2]** - PROMPTED a referral or investigation for surveillance/monitoring that MAY NOT BE NECESSARY (e.g. variant of uncertain significance) **[1]** - DID NOT PROMPT a referral/investigation for surveillance/monitoring **[0]** | |
| 1. Provided information to guide medication management | - GUIDED current medication management **[2]** - MAY GUIDE medication management in the future **[1]** - DID NOT PROVIDE information that would guide medication management, now or in the future **[0]** | |
| 1. Provided information about surgical management | - ENABLED a discussion or offer of a surgical option **[2]** - AVOIDED a discussion or offer of a surgical option **[1]** - A surgical option is NOT RELEVANT at this time or NOT RELATED to the genetic test results **[0]** | |
| 1. Provided information about a contraindicated behaviour (e.g. competitive sports) | - ENABLED me to provide information about a contraindicated behaviour **[2]** - Information about a contraindicated behaviour is NOT RELEVANT at this time **[0]** | |
| 1. Provided recurrence risk information for my patient | - Provided recurrence risk information that is RELEVANT to my patient at this time **[2]** - Provided recurrence risk information that MAY BE RELEVANT to my patient in the future **[1]** - Cannot be determined (e.g. variant of uncertain significance, did not provide information) **[0]** | |
| 1. Provided recurrence risk information for my patient’s family | - Provided recurrence risk information that is RELEVANT to my patient’s family at this time **[2]** - Provided recurrence risk information that MAY BE RELEVANT to my patient’s family in the future **[1]** - Cannot be determined (e.g. variant of uncertain significance, family member(s) did not receive testing or unknown if tested) **[0]** | |
| 1. Clarified potential health risks for my patient’s family | - CLARIFIED potential health risks for my patient’s family **[2]** - DID NOT CLARIFY health risks for my patient’s family **[0]** - Cannot be determined (e.g. variant of uncertain significance, family member(s) did not receive testing or unknown if tested) **[0]** | |
| 1. Generated psychosocial benefit for my patient or his/her family | - SIGNIFICANT psychosocial benefit was experienced **[2]** - MODERATE psychosocial benefit was experienced **[1]** - NO psychosocial benefit was experienced **[0]** - Cannot be determined **[0]** | |
| 1. Generated psychosocial concern for my patient or his/her family | - SIGNIFICANT psychosocial concern was experienced **[-2]** - MODERATE psychosocial concern was experienced **[-1]** - NO psychosocial concern was experienced **[0]** - Cannot be determined **[0]** | |

**Items 1-5:**  Not applicable when the proband is deceased. In this case, item should be left blank.

**Item 5:** The recurrence risk information is considered relevant if the patient is of reproductive age and is considering having children now or in the near future (i.e. within 1 year).

**Item 6:** Family includes parents, siblings and extended family. The recurrence risk information is considered relevant if the patient’s family member is of reproductive age and is considering having children now or in the near future (i.e. within 1 year).

**Item 7:** Reduction of risk counts. For example, if there was a question that other family members could have the same condition, but the primary finding was de novo, there would be a reduction of risk for a family member.

**Items 8/9:** If you do not have a clear memory of the session or did not record psychological response in clinic notes, choose the ‘cannot be determined’ response option.

**C-GUIDE: Pharmacogenomics Index**

Did you disclose PHARMACOGENOMIC results?

- Yes
- No

N.B. For the purpose of this index, pharmacogenomic results include those that are identified through a targeted pharmacogenomic analysis and could be relevant to medication management now or in the future.

If yes, please complete C-GUIDE once for the pharmacogenomic result(s) disclosed. For the purpose of this study, pharmacogenomic results are typically disclosed as a ‘cluster’ of variants to the patient or family.

| **Item** | **Response options** |
| --- | --- |
| ***The genetic testing that my patient had…*** | |
| 1. Provided information to guide medication management for my patient | - GUIDED current medication management **[2]** - MAY GUIDE medication management in the future **[1]** - DID NOT PROVIDE information that would guide medication management, now or in the future **[0]** |
| 1. Provided information to guide medication management for my patient’s family | - GUIDED current medication management for my patient’s family **[2]** - MAY GUIDE medication management for my patient’s family in the future **[1]** - DID NOT PROVIDE medication management information for my patient’s family, now or in the future **[0]** - Cannot be determined (e.g. variant of uncertain significance, family member(s) did not receive testing or unknown if tested) **[0]** |
| 1. Generated psychosocial benefit for my patient or his/her family | - SIGNIFICANT psychosocial benefit was experienced **[2]** - MODERATE psychosocial benefit was experienced **[1]** - NO psychosocial benefit was experienced **[0]** - Cannot be determined **[0]** |
| 1. Generated psychosocial concern for my patient or his/her family | - SIGNIFICANT psychosocial concern was experienced **[-2]** - MODERATE psychosocial concern was experienced **[-1]** - NO psychosocial concern was experienced **[0]** - Cannot be determined **[0]** |

**Item 1:** Not applicable when the proband is deceased. In this case, item should be left blank.

**Item 2:** Family includes siblings and extended family.

**Items 3/4:** If you do not have a clear memory of the session or did not record psychological response in clinic notes, choose the ‘cannot be determined’ response option.

**Global item**

| Taking into account all of the results you have just rated for this test, the genetic testing that my patient had | - Prompted better care for my patient or his/her family **[2]** - May prompt better care for my patient or his/her family in the future **[1]** - Did not change the care provided to my patient or his/her family **[0]** |
| --- | --- |
